# Integrative Proteome and Transcriptome Analyses Reveal the Metabolic Disturbance of the Articular Cartilage in Kashin–Beck Disease, an Endemic Arthritis

**DOI:** 10.3390/ijms26115146

**Published:** 2025-05-27

**Authors:** Lixin Han, Bolun Cheng, Jinyu Xia, Shiqiang Cheng, Xuena Yang, Feng Zhang

**Affiliations:** NHC Key Laboratory of Environment and Endemic Diseases, Collaborative Innovation Center of Endemic Disease and Health Promotion for Silk Road Region, School of Public Health, Health Science Center, Xi’an Jiaotong University, Xi’an 710061, China; shaanxiyqfk@163.com (L.H.); xjy0626@stu.xjtu.edu.cn (J.X.); chengsq0701@stu.xjtu.edu.cn (S.C.); smile940323@stu.xjtu.edu.cn (X.Y.)

**Keywords:** Kashin–Beck disease, osteoarthritis, proteome, transcriptome, multi-omics

## Abstract

The objective of this study was to elucidate the proteomic and transcriptomic alterations within the cartilage in Kashin–Beck disease (KBD) compared to a normal control. We conducted a comparison of the expression profiles of proteins, mRNAs, and lncRNAs via data-independent acquisition (DIA) proteomics and transcriptome sequencing in six KBD individuals and six normal individuals. To facilitate the functional annotation enrichment analysis of the differentially expressed (DE) proteins, DE mRNAs, and DE lncRNAs, we employed bioinformatic analysis utilizing Gene Ontology (GO) and the Kyoto Encyclopedia of Genes and Genomes (KEGG). Additionally, we conducted integration analysis of multi-omics datasets using mixOmics. We revealed a distinct proteomic signature, highlighting 53 DE proteins, with notable alterations in the pathways related to tryptophan metabolism and microbial metabolism. Additionally, we identified 160 DE mRNAs, with the functional enrichment analysis uncovering pathways related to RNA metabolism and protein splicing. Furthermore, our analysis of the lncRNAs demonstrated biological processes involved in protein metabolism and cellular nitrogen compound metabolic processes. The integrative analysis uncovered significant correlations, including the positive correlation between superoxide dismutase 1 (*SOD1*) and mitochondrial import receptor subunit TOM6 homolog (TOMM6), and the negative correlation between C-X9-C motif-containing 1 (*CMC1*) and succinate–CoA ligase [GDP-forming] subunit beta, mitochondrial (SUCLG2). Our results provide novel insights into the molecular mechanisms underlying KBD.

## 1. Introduction

Kashin–Beck disease (KBD) is a chronic, endemic osteochondropathy that primarily affects children and adolescents. This disease is characterized by significant pathological changes in the growth plate and articular cartilage, leading to necrosis of the chondrocytes, which disrupts normal endochondral ossification [1]. Consequently, this disruption results in a range of clinical manifestations, including growth retardation, joint deformities, and functional impairments of multiple joints [2,3]. Typically, the disease presents in children aged 3 to 12 years, with the symptoms becoming more pronounced as they age [4]. In advanced stages, KBD can lead to severe complications, including dwarfism and permanent disability, significantly impacting quality of life [5]. The pathogenesis of KBD is closely linked to genetic alterations, making the investigation of key genes and proteins essential for understanding the disease’s progression and for developing potential therapeutic strategies.

Recent research on KBD has increasingly focused on transcriptomic analyses to elucidate the underlying biological mechanisms, particularly in relation to osteoarthritis (OA). For instance, RNA sequencing has highlighted differences in the gene expression profiles between KBD with OA and rheumatoid arthritis (RA), identifying key pathways involved in cartilage metabolism and degradation [6,7,8,9]. While mRNAs are traditionally the focus of transcriptomic studies, lncRNAs are increasingly recognized as pivotal regulators of gene expression at the transcriptional, post-transcriptional, and epigenetic levels [10]. Emerging evidence supports lncRNAs playing a key role in cartilage degradation and skeletal diseases [11]. However, a notable limitation of the existing research is the lack of normal controls, which limits comprehensive understanding of the disease mechanisms. The integration of transcriptomic and proteomic data remains insufficient, underscoring the necessity of combining multi-omics analyses to explore the mechanisms of KBD.

DIA proteomics has emerged as a powerful technique in the field of large-scale proteomics, offering significant advantages over traditional data-dependent acquisition (DDA) methods [12,13]. The high reproducibility and sensitivity of DIA make it particularly suitable for analyzing small amounts of clinical tissue specimens [14]. For bone-related diseases, DIA has been effectively applied to investigate the proteomic landscape of the cartilage and subchondral bone, providing insights into disease pathogenesis. For instance, a study utilizing DIA mapped the cartilage and subchondral bone proteomes, identifying multiple key proteins involved in osteogenesis and bone remodeling [15]. However, comprehensive studies utilizing DIA to compare the proteomes of KBD and normal control cartilage tissues remain lacking.

In this study, we employed DIA proteomics and RNA sequencing (RNA-seq) to profile the proteomic and transcriptomic alterations in KBD by comparing the joint cartilage of KBD patients to that of normal controls. Our integrated analysis revealed DE mRNAs, lncRNAs, and proteins, elucidating their functional implications. These findings enhance our molecular understanding of KBD and provide a foundation for developing targeted therapeutic strategies based on these biomarkers.

## 2. Results

### 2.1. Proteomic Profiles of Cartilage from Patients with and Without KBD

**Figure 1:**
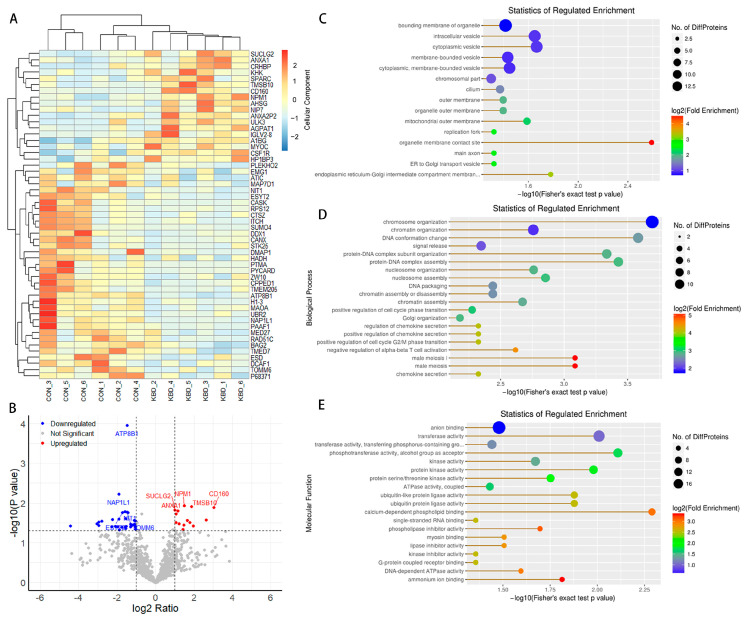
Expression profiles of proteins. Overview of the protein expression (KBD *n* = 6, control *n* = 6). (**A**) The heatmap is used to assess the expression of the proteins. Each DE protein is represented by a single row of colored boxes, and each sample is represented by a single column. (**B**) Volcano plots of the proteins. Red and blue denote high and low expression, respectively (*p* value < 0.05 and |log_2_FC| > 1). (**C**–**E**) CC, BP, and MF GO term analysis of the DE proteins (Fisher’s exact test *p* value < 0.05).

Our comparative proteomic analysis revealed a distinct proteomic signature between KBD and normal cartilage (Figure 1A), highlighting 53 proteins with significant differential expression (Supplementary Table S1). Among these, 18 proteins were upregulated, while 35 were downregulated (Figure 1B). For example, ATP8B1, implicated in phospholipid transport; A1BG, known for its acute-phase protein properties; SUCLG2, a key enzyme in the citric acid cycle; ANXA1, an anti-inflammatory protein; and TOMM6, a component of the mitochondrial import machinery. Three pathways emerged as significantly altered: tryptophan metabolism, microbial metabolism in diverse environments, and biosynthesis of secondary metabolites (Supplementary Table S2). Furthermore, our analysis identified 15 significant GO terms under the cellular component (CC) (Figure 1C), including organelle membrane contact sites and cytoplasmic vesicles, suggesting alterations in intracellular trafficking and organelle interactions. Under the biological process (BP) (Figure 1D), 117 significant GO terms were noted, such as chromosome organization, DNA conformation changes, and protein–DNA complex assembly, which may suggest disruptions to cellular processes and genetic regulation. For the molecular function (MF) (Figure 1E), 19 significant GO terms were highlighted, focusing on calcium-dependent phospholipid binding and various enzymatic activities, reflecting changes in signal transduction and metabolic regulation (Supplementary Table S2).

### 2.2. mRNA Differences in Cartilage from Patients with and Without KBD

**Figure 2:**
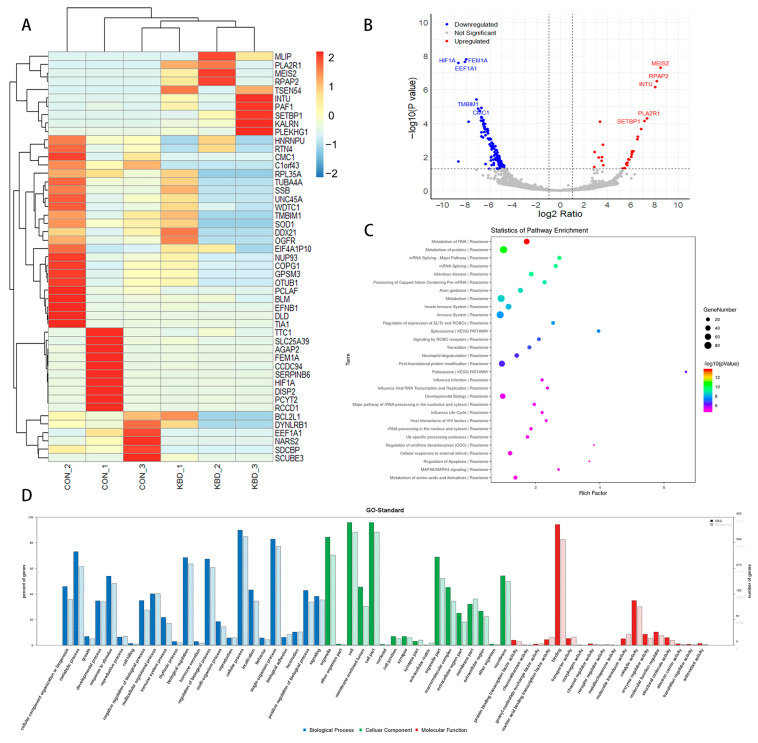
Expression profiles of mRNAs. Overview of the mRNA expression (KBD *n* = 3, control *n* = 3). (**A**) The heatmap is used to assess the expression of the mRNAs. Each DE mRNA is represented by a single row of colored boxes, and each sample is represented by a single column. (**B**) Volcano plots of the mRNAs. Red and blue denote high and low expression, respectively (*p* value < 0.05 and |log_2_FC| > 1). (**C**) Pathway analysis of the DE mRNAs (Fisher’s exact test *p* value < 0.05). (**D**) GO term analysis of the DE mRNAs (Fisher’s exact test *p* value < 0.05).

Our study revealed significant transcriptional differences between the KBD and control samples (Figure 2A), with 158 DE mRNAs identified (Supplementary Table S3), of which 30 were upregulated and 128 were downregulated in KBD (Figure 2B). The functional enrichment analysis of these DE mRNAs uncovered 226 significant pathways (Figure 2C, Supplementary Table S4), including the metabolism of RNA, metabolism of proteins, mRNA splicing—major pathway, and infectious disease. Furthermore, 316 significant GO terms under BP were enriched, highlighting processes such as mRNA metabolic process, RNA splicing, and cellular macromolecule catabolic process. Under CC, 79 significant GO terms were identified, including the intracellular organelle part and cytoplasm. For MF, 32 significant GO terms were enriched, focusing on protein binding, RNA binding, and nucleic acid binding (Figure 2D, Supplementary Table S4). To assess the transcriptomic–proteomic concordance, we examined the mRNA fragments per kilobase of transcript per million mapped reads (FPKM) of genes encoding the top five DE proteins. Although these mRNAs did not reach statistical significance, directional trends were observed. For instance, both *TMSB10* and *ANXA1* exhibited upregulation in the KBD samples at both the RNA and protein levels (Supplementary Figure S1).

### 2.3. Differential lncRNA Expression and Target Gene Prediction and Functional Analysis

**Figure 3:**
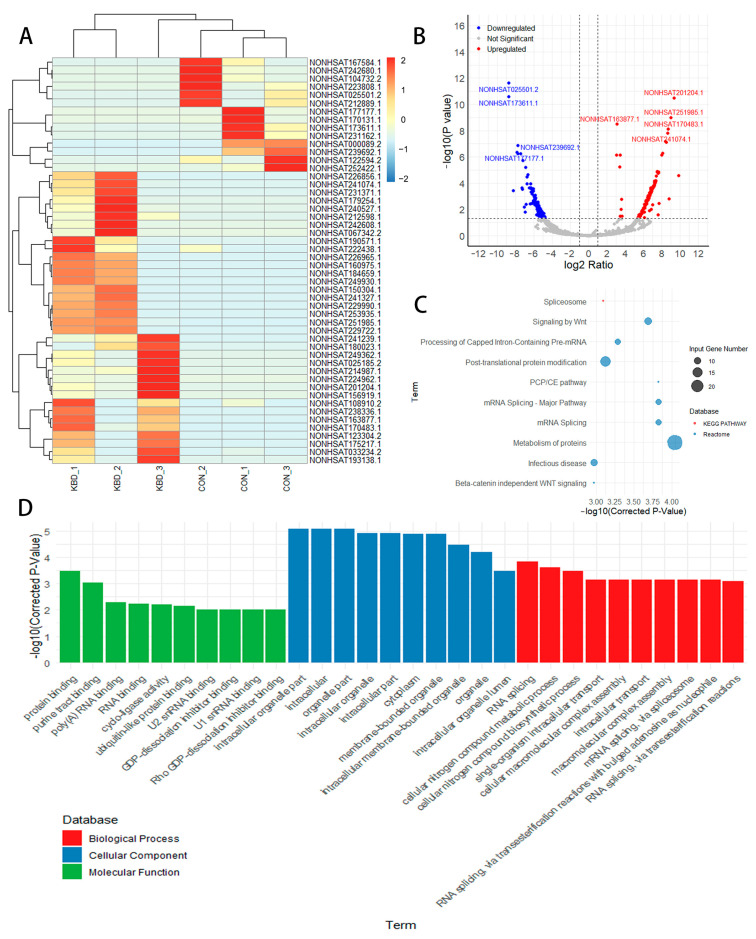
Expression profiles of long intergenic ncRNAs. (**A**) The heatmap is used to assess the expression of the lncRNAs. (**B**) Volcano plots of the lncRNAs. (**C**) Pathway analysis of the target genes of the DE lncRNAs. (**D**) GO analysis of the target genes of the DE lncRNAs.

Our analysis revealed a significant alteration in the lncRNA landscape between the KBD and control samples (Figure 3A), with 218 lncRNAs exhibiting differential expression—111 being upregulated and 107 downregulated in KBD (Figure 3B, Supplementary Table S5). To identify potential functional targets of the DE lncRNAs, we employed BLAST2GO to predict the target genes through sequence homology analysis. We identified 60 lncRNAs that target DE genes (Supplementary Table S6). For the predicted target genes of the DE lncRNAs, a total of 192 significant pathways were enriched (Figure 3C, Supplementary Table S7), such as the metabolism of proteins, mRNA splicing, and signaling by Wnt. Under the GO categories, we identified 170 significant BP terms, including RNA splicing and cellular nitrogen compound metabolic process. For CC, 79 significant terms were enriched, such as the intracellular organelle part and cytoplasm. For MF, 19 significant terms were identified, including protein binding and RNA binding (Figure 3D, Supplementary Table S7).

### 2.4. Functional Enrichment Comparison Among Multi-Omics Data

Due to the lack of overlap between the RNA and protein data, we investigated whether there was overlap at the pathway or ontology level. The tri-omics (lncRNA–mRNA–protein) pathway and ontology overlap included two GO terms, metabolic process and cellular macromolecular complex assembly. These GO terms reflect core cellular processes critical for maintaining cartilage homeostasis. The mRNA–protein overlap included eight GO terms, such as secretion by cell, cell cycle G2/M transition, and RNA biosynthetic process. The lncRNA–protein overlap included one GO term, single-stranded RNA binding. lncRNAs may regulate protein activity by binding RNA-binding proteins. The lncRNA–mRNA overlap included 135 GO terms, such as immune/inflammatory regulation, RNA metabolic process, intracellular organelle, and regulation of macromolecule metabolic process (Supplementary Table S8). The lncRNA–mRNA overlap included 123 pathways, such as the metabolism of proteins, regulation of apoptosis, and MAPK family signaling cascades (Supplementary Table S9). The extensive lncRNA–mRNA overlap highlights the transcriptional and post-transcriptional regulatory networks in KBD.

### 2.5. Integrative Analysis of Proteome and Transcriptome

**Figure 4:**
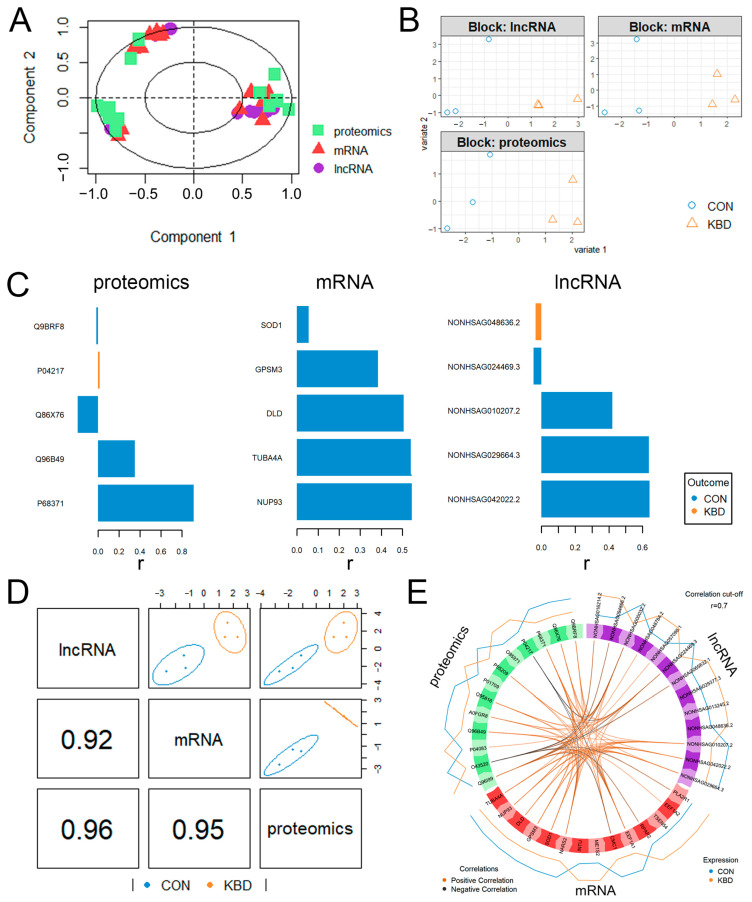
Sparse partial least squares discriminant analysis. (**A**) Correlation circle plot for the combination of the three datasets. The variable types are indicated with different symbols and colors, overlaid on the same plot. (**B**) The individual contribution of each dataset, showing the score plots for the first two components, indicating the efficient separation capability for the three datasets. The samples are plotted according to their scores for the first two components for each dataset, colored by KBD or control. (**C**) Top 5 selected features shown in the pyramid bar plot. The most important variables (according to the absolute value of their coefficients) are ordered from bottom to top. As this is a supervised analysis, the colors indicate the class for which the median expression value is the highest for each feature. The X-axis represents the correlation coefficient (r) between the molecule and KBD or the control. (**D**) Sample scatterplot from plotDiablo displaying the first component in each dataset (upper diagonal plot) and the Pearson’s correlation between each component (lower diagonal plot). The samples are colored by KBD or control, with the 95% confidence ellipse plots represented. (**E**) The Circos plot (cutoff: 0.7) shows positive or negative correlations greater than 0.7 between variables of different types, denoted as red and blue lines, respectively.

Figure 4A shows the correlation of the top 20 features in the three datasets. The first component of the sparse partial least squares discriminant analysis (sPLS-DA) [16] of the combined proteome and transcriptome datasets clearly discriminated the normal from the KBD cartilage samples (Figure 4B). This filtering identified proteins, mRNAs, and lncRNAs that distinguished KBD from the normal control samples (Figure 4C). For example, Q9BRF8 (serine/threonine-protein phosphatase CPPED1), P04217 (alpha-1B-glycoprotein), Q86X76 (deaminated glutathione amidase), Q96B49 (mitochondrial import receptor subunit TOM6 homolog), and P68371 (tubulin beta-4B chain) were highlighted. For example, P04217 (alpha-1B-glycoprotein) and NONHSAG048636.2 were KBD-specific, which may potentially distinguish the KBD samples from the controls. The DIABLO method identified several important features discriminating KBD from the normal controls by interrogating the correlations between the three omics datasets, with the lncRNA and proteome data showing the highest correlations (Pearson’s r = 0.96) (Figure 4D). Finally, to visualize the between-omics correlations in the DIABLO analysis, a Circos plot revealed numerous strong positive and negative correlations (Pearson’s r > 0.70) (Figure 4E). For example, *SOD1* was positively correlated with Q96B49 (TOMM6). *CMC1* was negatively correlated with Q96I99 (SUCLG2) and positively correlated with P68371 (tubulin beta-4B chain).

## 3. Discussion

In this study, we established the proteomic and transcriptomic profiles of the articular cartilage in KBD and normal control samples, aiming to elucidate the molecular mechanisms of KBD. Our comprehensive analysis included proteins, lncRNAs and mRNAs, and it identified complex network of features and gene–protein interactions, offering novel insights into KBD’s etiology.

Our findings identified metabolic and oxidative stress pathways as central to KBD’s pathogenesis. For example, low selenium levels and T-2 toxin exposure have been implicated in cartilage damage through mechanisms involving mitochondrial dysfunction and reactive oxygen species (ROS) accumulation [17]. Our multi-omics data corroborated this and revealed that downregulation of *SUCLG2* (succinate-CoA ligase) and upregulation of *SOD1* align with the mitochondrial energy metabolism defects and compensatory antioxidant responses observed in KBD chondrocytes [18]. In addition, the enrichment of the microbial metabolism pathways suggests a potential link between gut microbiota dysbiosis and KBD progression, a hypothesis supported by a recent genomic study [19]. However, our study provides novel insights by integrating proteomic and transcriptomic data to identify the lncRNA-mediated regulation of protein metabolism. While both KBD and OA involve cartilage degradation, our findings highlight key molecular distinctions. OA is primarily driven by mechanical stress and age-related inflammation (e.g., IL-1β, TNF-α) [20], whereas KBD exhibits early-onset mitochondrial dysfunction and a selenium-dependent redox imbalance [21]. In OA, lncRNAs such as plasmacytoma variant translocation 1 (*PVT1*) regulate chondrocyte apoptosis and matrix metalloproteinase (MMP) expression [22]. In contrast, our study identifies KBD-specific lncRNAs enriched in nitrogen compound metabolism.

The transcriptome and proteomics analysis of KBD found that there were no overlapping molecules. This finding indicated the potential roles of post-transcriptional regulation and epigenetic regulation in this process. Firstly, the absence of overlapping genes between the transcriptome and the proteome is not uncommon. Research has indicated that the regulation of gene expression is influenced not only by the transcription level but also by various factors, including post-transcriptional modification, post-translational modification and protein degradation. For example, numerous genes may be regulated by lncRNAs and other mechanisms after transcription [23]. Secondly, epigenetic alterations may contribute to the inconsistencies observed between the transcriptome and the proteome. Epigenetic modifications such as DNA methylation and histone modification can regulate gene expression without altering the DNA sequence. These alterations may be crucial to the pathogenesis of KBD, as certain epigenetic modifications have been strongly linked to the onset and progression of arthritis [24]. Additionally, the intricate environmental risk factors associated with KBD may complicate the identification of key genes. Variations in these processes can influence gene expression patterns, resulting in differences between the transcriptomic and proteomic profiles.

Our integrated omics analysis has revealed a range of proteins and transcripts related to metabolism that could serve as potential biomarkers for KBD. For instance, ANXA1, a protein known for its anti-inflammatory and immunomodulatory functions, has been associated with the resolution of inflammation in arthritic conditions [25]. TMSB10, recognized for its contributions to angiogenesis and osteogenesis, has been applied in bone tissue engineering to enhance bone regeneration [26]. SUCLG2, which is part of the citric acid cycle, has been shown to be upregulated by fibroblast growth factor 2 (*FGF2*) in osteoblasts, highlighting its significance in relation to bone development and metabolism [27]. The differential expression of these proteins in KBD implies that they may have crucial impacts on cartilage metabolism. Upon differential gene expression analysis, we noticed many DEGs were actually lncRNAs and therefore decided to investigate the target genes of these lncRNAs. An RNA-seq study identified 4103 DE lncRNAs in the KBD cartilage, implicating pathways such as Wnt signaling, lysosome function, and TNF signaling [11]. These pathways align with our proteomic findings, suggesting the lncRNAs may bridge the transcriptional and metabolic dysregulation in KBD. In OA, the lncRNAs regulate chondrocyte apoptosis and matrix degradation [28]. Similarly, our study identified lncRNAs enriched in protein metabolism and nitrogen compound processes, potentially suggesting these regulatory roles in KBD.

Our enrichment analyses revealed that several functions within bone- and cartilage-related pathways may be critical to KBD’s pathogenesis. The enrichment analysis of the DE proteins underscored the potential metabolic dysregulation associated with KBD, particularly concerning tryptophan metabolism, which has been linked to inflammatory responses [29]. Moreover, the identified GO biological processes suggested that changes in genomic stability and transcriptional regulation may play a role in the disease’s progression [30]. The enrichment analysis of the DE mRNAs highlighted pathways related to RNA metabolism and splicing, as well as immune system functions. The involvement of mRNA splicing pathways emphasized the significance of post-transcriptional regulation in KBD, which may influence the expression of key inflammatory mediators [31]. Additionally, the GO biological processes associated with cellular nitrogen compound metabolism further highlighted the metabolic alterations occurring in KBD, potentially affecting cellular homeostasis and immune responses. The identification of RNA splicing and intracellular transport processes among the enriched GO terms for the lncRNA targets suggested that the lncRNAs may modulate the expression and function of proteins involved in these essential pathways. The tri-omics overlaps highlighted metabolic pathways vulnerable to KBD stressor selenium deficiency. The tri-omics enrichment of the metabolic pathways supports the hypothesis that mitochondrial failure underlies KBD chondrocyte degeneration. In addition, the immune pathways, such as NF-κB, overlapped in the lncRNAs and mRNAs suggested inflammation progress in KBD. Collectively, these findings indicate that immune responses, alterations in the endocrine system, and metabolic changes represent the primary functional shifts observed between KBD and controls.

The integration of data through multi-omics approaches is essential for studies on bone and cartilage metabolism [32]. Our integrative analysis has provided a comprehensive perspective on the molecular alterations in KBD patients, emphasizing the association between the proteome and the transcriptome in regulating phenotypes [33]. We identified numerous proteins, mRNAs, and lncRNAs that differentiate KBD from normal controls. The *SOD1* gene, a crucial component of the oxidative stress response, showed positive correlations with TOMM6, a subunit of the mitochondrial import receptor. Previous studies have indicated that *SOD1* is vital to managing oxidative stress [34,35] and maintaining bone homeostasis [36]. The upregulation of *SOD1* and downregulation of glutathione-related enzymes suggested that boosting the endogenous antioxidant defenses (e.g., N-acetylcysteine [37], selenium-rich diets [38]) may mitigate the oxidative damage in KBD cartilage. Moreover, the microbial metabolism pathway enrichment suggested that probiotics or dietary modifications to reduce T-2 toxin exposure may disrupt KBD progression, aligning with the fungal toxin hypothesis of KBD’s etiology [39].

Our research utilized high-throughput technologies to characterize KBD from various perspectives, encompassing both proteomic and transcriptomic analyses. DIABLO provides a robust statistical framework for integrating disparate datasets—comprising RNA and protein data. This approach offers a comprehensive view of the molecular landscape of KBD. However, there are several limitations and future directions. Firstly, the modest sample size may reduce the statistical power to detect subtle transcriptomic and proteomic differences. While our multi-omics integration approach partially mitigates this issue by prioritizing pathways across datasets, future studies with larger cohorts are needed to validate these findings. Secondly, while this study focused on multi-omics discovery, functional validation of the prioritized targets using in vitro or in vivo models is essential to confirm their causal roles in KBD’s pathogenesis. Thirdly, it is difficult to correlate molecular signatures with clinical outcomes due to the small cohort size and limited clinical metadata. Future studies integrating multi-omics profiling with detailed phenotyping in larger KBD cohorts are essential.

In summary, our study presents a comprehensive analysis of the transcriptional and proteomic alterations in KBD, highlighting the potential of integrated omics approaches in unraveling the complexities of the disease pathology. By examining the functional pathways and differential expression of RNAs and proteins, we identified key genes and non-coding RNAs that may provide novel insights into KBD’s etiology.

## 4. Materials and Methods

### 4.1. Ethics Statement

This study was approved by the Human Ethics Committees of Xi’an Jiaotong University. Written informed consent was obtained from all the study participants.

### 4.2. Samples

**Figure 5:**
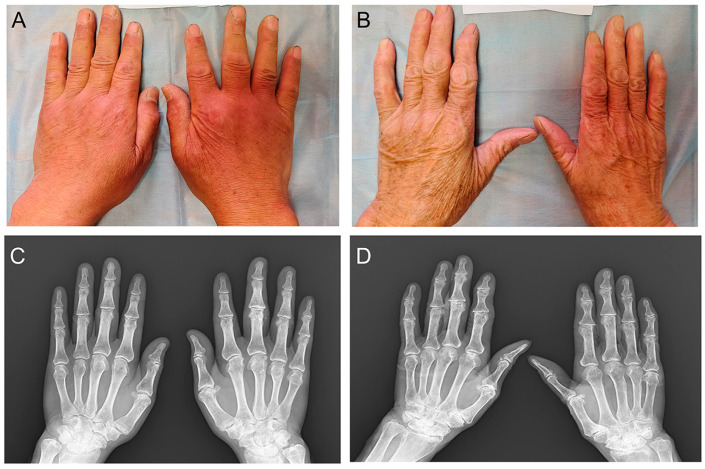
Characteristics of the study samples with Kashin–Beck disease. (**A**) Hand images of male patient; (**B**) hand images of female patient; (**C**) hand X-ray of male patient; and (**D**) hand X-ray of female patient. The image (**A**) and X-ray (**C**) are from the same patient, while the image (**B**) and X-ray (**D**) are from the same patient.

Cartilage specimens were obtained from the knee joints of patients diagnosed with KBD and from age- and sex-matched control subjects. The diagnosis of KBD was established based on clinical evaluations and imaging studies, adhering to the KBD diagnosis criterion of China (WS/T 207-2010 [40], https://www.ndcpa.gov.cn/jbkzzx/c100200/common/content/content_1666632341665943552.html accessed on 25 May 2025). Participants with a history of genetic bone and cartilage disorders, RA, or other significant joint diseases were excluded from this study. The KBD group included six individuals (three males and three females) aged between 54 and 75 years (Figure 5), while the control group comprised six subjects (three males and three females) aged between 57 and 71 years. For the proteomic analysis, a total of six KBD and six control samples underwent differential protein expression analysis using DIA liquid chromatography–mass spectrometry (LC-MS). Additionally, RNA sequencing was performed on three KBD samples and three control samples to investigate the gene expression profiles. The average ages of the KBD and control groups were 66.5 ± 7.21 and 63.5 ± 5.12 years, respectively.

### 4.3. Proteomic Analysis

#### 4.3.1. Sample Preparation

For the proteomic analysis, cartilage samples from KBD patients and control subjects were processed following a standardized protocol. Initially, proteins were extracted from the cartilage tissues using a lysis buffer containing 8 M urea and 50 mM ammonium bicarbonate. The protein concentration was determined using the Bradford assay, ensuring accurate quantification prior to further processing. Subsequently, the extracted proteins underwent enzymatic digestion with trypsin at 37 °C for 16 h, allowing for the generation of peptides suitable for mass spectrometry analysis. Following digestion, the samples were desalted using C18 solid-phase extraction to remove any contaminants and concentrate the peptides. Finally, the purified peptides were reconstituted in a suitable buffer for library construction and mass spectrometry analysis.

#### 4.3.2. LC-MS Analysis

The prepared peptide samples were subjected to LC-MS (Shimadzu, Inc., Shanghai, China) for comprehensive proteomic profiling. The samples were loaded onto a C18 column and separated using a gradient elution method. Mass spectrometry was performed on a Q Exactive mass spectrometer (ThermoFisher, Inc., Shanghai, China), operating in both DDA and DIA modes. The DDA mode was utilized for spectral library generation, while the DIA mode allowed for the quantification of proteins across the samples. The mass spectrometer settings included a resolution of 70,000 for the MS1 scans and 17,500 for the MS2 scans, with a scan range of 300 to 1500 *m*/*z*. The data generated were processed to identify and quantify proteins based on their peptide sequences.

#### 4.3.3. DIA Proteomics Analysis

To facilitate the analysis of the mass spectrometry data, a customized spectral library was constructed. The raw data files obtained from the LC-MS analysis were processed using MaxQuant (v2.0.0), which enabled the identification of peptides and proteins based on the UniProt protein database. The parameters for the database searching included trypsin digestion with a maximum of two missed cleavages and a mass tolerance of 20 ppm for precursor ions. The false discovery rate (FDR) for the protein identification was set at 1%. The searching result was exported in a .tsv file format containing the annotation of the precursors and fragment ions and their exact retention times. The .tsv file was then imported into DIA-NN (v1.8.1) to generate the spectral library used for the DIA data analysis. The DIA data were analyzed with DIA-NN, a mass spectrometer vendor-independent free software for DIA data analysis. The raw data were analyzed according to the user guide for the software, with default settings established for the protein identification and peak area calculation. A target-decoy-based strategy was applied to control the FDR at lower than 1%. A *p* value < 0.05 and |log_2_FC| > 1 were set as cutoffs to identify significantly dysregulated proteins.

#### 4.3.4. Functional Enrichment Analysis

The biological significance of the DE proteins was assessed through enrichment analysis. GO enrichment analysis was performed to categorize the proteins based on their BP, MF, and CC, implemented using the GOseq (v3.21) [41]. Additionally, KEGG pathway analysis was conducted to identify the pathways associated with the DE proteins, utilizing KOBAS (v3.0) [42]. The enrichment was considered significant for GO terms and KEGG pathways with a corrected *p* value of less than 0.05.

### 4.4. Transcriptomic Analysis

#### 4.4.1. RNA Extraction, Library Preparation, and RNA-Sequencing

The total RNA was isolated and purified using TRIzol reagent (Invitrogen, Carlsbad, CA, USA) following the manufacturer’s procedure. A targeted approach for RNA extraction was employed, focusing on the removal of ribosomal RNA (rRNA) to enrich the mRNA and long non-coding RNA (lncRNA). This process was facilitated using the Ribo-Zero™ rRNA Removal Kit (Illumina, San Diego, CA, USA). The subsequent library preparation was carried out following the manufacturer’s protocol, and the library was sequenced on the Illumina HiSeq 4000 platform (Illumina, Inc., San Diego, CA, USA), utilizing paired-end sequencing technology. The post-sequencing data processing involved a series of bioinformatics tools, including Cutadapt (v5.0) [43] for adapter trimming and quality control, FastQ (v0.14.0) for sequence quality assessment (http://www.bioinformatics.babraham.ac.uk/projects/fastqc/, accessed on 10 January 2025), Bowtie2 (v2.5.2) [44] for aligning the reads to the reference genome, Hisat2 (v2.1.0) [45] for mapping the reads to the genome of hg38, StringTie (v1.3.3) [46] for transcript assembly and quantification, and edgeR (v3.18.1) [47] for estimating the expression levels of all the transcripts.

#### 4.4.2. LncRNA Identification

Transcripts that overlapped with known mRNAs and those shorter than 200 bp were discarded. We utilized CPC (v0.9r2) [48] and CNCI (v2.0) [49] to predict transcripts with coding potential. All the transcripts with a CPC score of <−1 and a CNCI score of <0 were removed, and the remaining transcripts were considered lncRNAs. We focused on long intergenic non-coding RNAs, defined as transcripts of >200 nucleotides lacking protein-coding potential, to investigate their regulatory roles in KBD.

#### 4.4.3. Different Expression Analysis of mRNAs and lncRNAs

StringTie [46] was used to perform the expression level calculations for the mRNAs and lncRNAs by calculating the FPKM [50]. The DE mRNAs and lncRNAs were selected with |log_2_FC| > 1, with statistical significance defined as an adjusted *p* value < 0.05, using the R package edgeR (v3.18.1) [47].

#### 4.4.4. Target Gene Prediction and Functional Analysis of lncRNAs

To elucidate the functional implications of the identified lncRNAs, we employed BLAST2GO (v6.0.1) [51] for the prediction of potential target genes. This tool enables the functional annotation of genes based on the sequence homology, providing insights into the potential biological roles of lncRNAs. By focusing on genes in proximity to lncRNAs, we aimed to uncover the cis-regulatory mechanisms that may influence the gene expression patterns. Significant differences in gene expression were assessed with |log_2_FC| > 1 and *p* < 0.05, followed by GO and pathway analysis. Enrichment analysis was performed to calculate the *p* value by Fisher’s exact test, with *p* < 0.05 set as the significance threshold.

### 4.5. Integration Analysis of Multi-Omics Datasets

To uncover the intricate relationships and novel insights that span different molecular levels, we conducted an integrated analysis using mixOmics (v6.10.9) [52], which facilitates the integration of heterogeneous omics data through multivariate statistical approaches. Our analysis focused on 3 KBD and 3 healthy controls. From each of the three omics layers—proteomics, mRNA, and lncRNA—we selected the top 20 features based on their statistical significance. The integration process leveraged the Data Integration Analysis for Biomarker discovery using DIABLO framework [53], which is embedded within the mixOmics package (v6.10.9).

### 4.6. Data and Software Availability

The original contributions of this study are provided in the Supplementary Material.

### 4.7. Statistical Analysis

All the statistical analyses were performed by using R software (version 4.3.0). All the results are presented as the mean ± standard deviation (SD), and a *p* value < 0.05 was considered statistically significant in our study.

## Data Availability

All the transcriptomic (RNA-seq) and proteomic (DIA-MS) data generated in this study have been deposited in the figshare repository under accession https://doi.org/10.6084/m9.figshare.29060792.v1.

## Supplementary Materials

The following supporting information can be downloaded at https://www.mdpi.com/article/10.3390/ijms26115146/s1.
