# Supplementary figures and images for: Integrative Proteome and Transcriptome Analyses Reveal the Metabolic Disturbance of the Articular Cartilage in Kashin–Beck Disease, an Endemic Arthritis
Source: Int J Mol Sci. 2025 May 27;26(11):5146. doi: 10.3390/ijms26115146 (PMC12155242; doi:10.3390/ijms26115146)

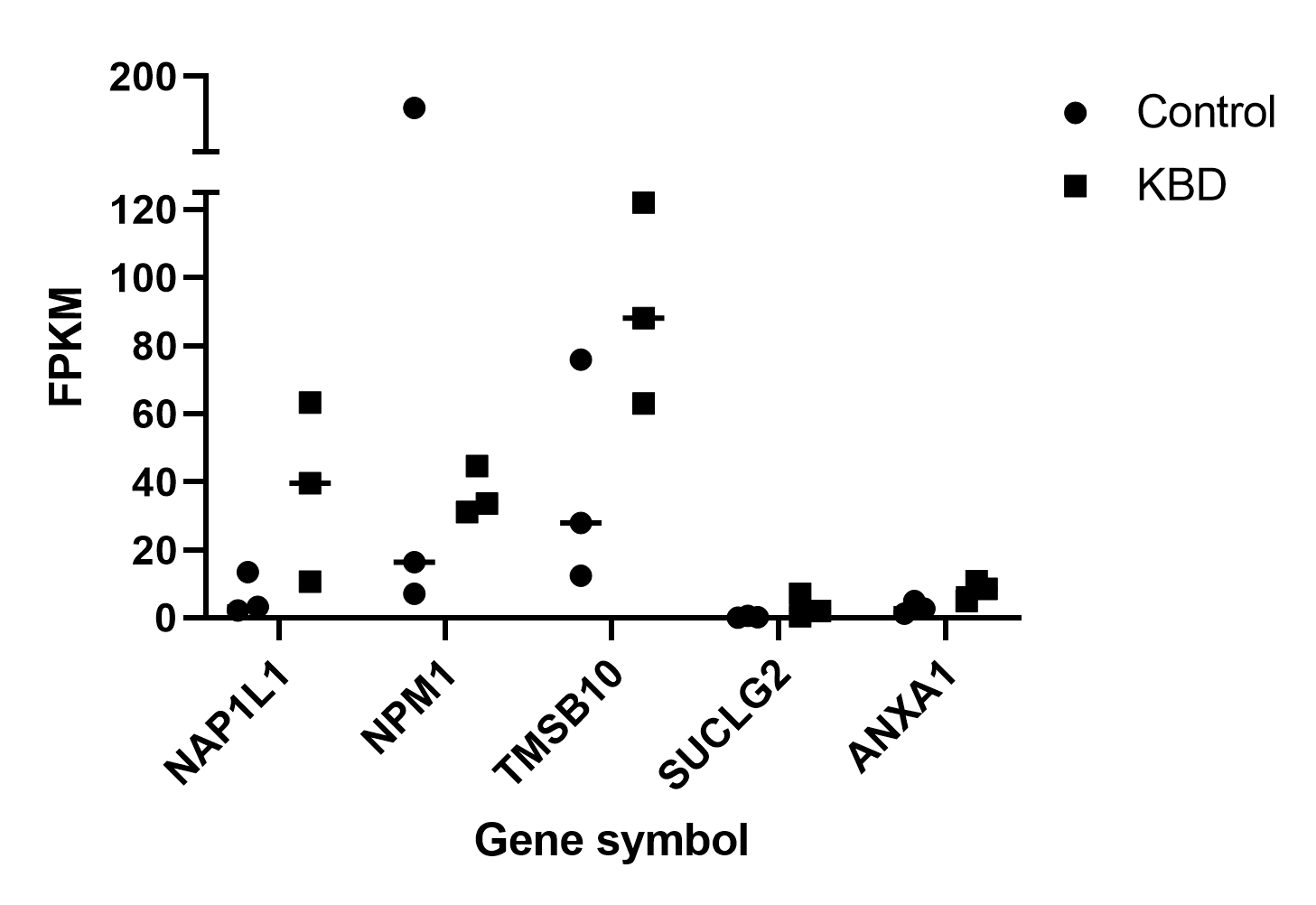

Supplement: Supplementary file 1 [file ijms-26-05146-s001.zip › Supplementary Figure S1.tif]
